# Improved cognitive flexibility after high-intensity interval training (HIIT) in individuals with Parkinson's disease: a pilot randomized controlled trial

**DOI:** 10.3389/fresc.2026.1835182

**Published:** 2026-08-05

**Authors:** André Hürlimann, Manuela Pastore-Wapp, Tim Vanbellingen

**Affiliations:** 1 Neurocenter, Luzerner Kantonsspital, Luzern, Switzerland; 2 Faculty of Health Science and Medicine, University of Lucerne, Lucerne, Switzerland; 3 Gerontechnology and Rehabilitation Group, ARTORG Center for Biomedical Engineering Research, University of Bern, Bern, Switzerland; 4 VITREA Schweiz AG, Bern, Switzerland

**Keywords:** cognitive flexibility, executive function, high-intensity interval training, Parkinson's disease, rehabilitation

## Abstract

**Background:**

People with Parkinson's disease (PwPD) commonly experience impairments in both motor and cognitive domains, particularly executive function and cognitive flexibility. While aerobic exercise may benefit non-motor domains, it remains unclear whether high-intensity interval (HIIT) provides superior cognitive benefits compared to intensive continuous endurance training (CT). This randomized pilot study investigated whether HIIT on a stationary bike leads to greater improvements in cognitive performance in PwPD than high-intensity endurance training on a stationary bike, with the aim of providing preliminary evidence of efficacy for a larger-scale RCT.

**Methods:**

Twenty people with Parkinson's disease were randomly assigned to either an HIIT group or a control group that performed CT. Both interventions were performed on a stationary cycle ergometer three times per week for three weeks (30 min/session), matched for overall training duration. HIIT consisted of alternating 30-second intervals at 75% and 90% of maximum heart rate, while the control group trained continuously at 85% of maximum heart rate. Primary outcomes included executive function such as cognitive flexibility and inhibitory control assessed by the Trial Making Test (TMT) and Stroop tests. Secondary outcomes comprised aerobic capacity (VO_2_ max), balance (Mini-BESTest), motor parkinsonian symptoms (Unified Parkinson Disease Rating Scales II-III), freezing of gait, and quality of life (Parkinson Disease Questionnaire-39). Pre-and post-intervention assessments were compared using 2 × 2 ANOVA and paired t-tests.

**Results:**

All participants completed the intervention. The HIIT group demonstrated significant within-group improvements in TMT-B (*p* = 0.010) and Stroop III (*p* = 0.019) indicating enhanced cognitive flexibility and inhibitory control, whereas no significant changes were observed in the control group. The TMT-B test revealed a significant interaction effect between the groups (*p* = 0.003, F = 12.716, and p*η*^2^ = 0.459). Both groups showed significant improvements in balance and motor function (both *p* < 0.001). VO2max increased significantly only in the CT group (*p* = 0.010). No differences were found for quality of life or freezing of gait (*p* = 0.530).

**Discussion and conclusions:**

HIIT on a cycle ergometer is feasible and well tolerated in PwPD and may preferentially enhance executive function such as cognitive flexibility and inhibitory control compared with CT over a short intervention period. Although exploratory, these findings suggest HIIT could be a promising strategy targeting cognitive deficits in PwPD. However, larger well-powered trails with longer follow-up are warranted to confirm these preliminary results and clarify underlying mechanisms.

## 1 Introduction

PD is characterized by its cardinal symptom, bradykinesia in combination with at least one of the following: tremor, rigidity or postural instability (1). People with Parkinson's disease (PwPD) often exhibit reduced performance across both physical and cognitive domains (5).

In addition to mood, behavior, speech, depression, anxiety, and apathy, cognitive impairment, for example, bradyphrenia, verbal memory, divided attention, executive functions, is one of the non-motor symptoms of PD and is an integral component of the disease's natural course. Impaired executive function, in particular, becomes apparent as early as Hoehn and Yahr Stages I–III (2). Parkinson's dementia often does not develop until the later stages of the disease (3). These non-motor symptoms affect 30%–50% of PwPD (4, 5) and endurance training has been demonstrated to improve some of these symptoms (6). Evidence suggests that exercise can positively influence the prognosis of PwPD (7). In addition, the benefits of moderate aerobic interval training for cognitive abilities have also been demonstrated, but a comparison with high-intensity interval training (HIIT) is still lacking (8, 9). It is hypothesized that HIIT may also lead to improvements, as positive neuroplastic changes triggered by elevated levels of brain-derived neurotrophic factor (BDNF) have already been documented (10). Currently, other biomarkers in the blood that could trigger these positive effects following HIIT are also being discussed, although their exact role remains unclear (11). Cardiorespiratory improvements resulting from HIIT in PwPD have also been demonstrated (12).

There is currently no standard definition for the duration and intensity of intervals in HIIT. Some studies have examined 15-second repetition intervals (13), while others have used 6-minute intervals (14). Since shorter phases allow for a higher number of intensity changes in the same amount of time, this study uses the relatively short 30-second intervals (15). To achieve the desired intensities for HIIT and CT training methods, the target heart rate for the workout is calculated, for example, based on the maximum heart rates (HRmax) determined during spiroergometric tests. Previous studies have shown that people with Parkinson's disease achieve a comparable HRmax during training as healthy individuals (16). This HRmax was therefore used to determine the training heart rate. Alternatively, the maximum oxygen uptake capacity (VO2max), which is also determined in a spiroergometric test, can be used. In some studies on HIIT, training intensity is also defined using the term “all-out,” meaning that during the high-intensity phase, the workout is performed at the participant's perceived maximum intensity (17). In this study, HRmax is used as a training parameter because it is easy to apply and yet can be measured accurately; VO2max is used here to quantify the training effect. To vary the training intensity, this study adjusts the watts (pedal resistance).

Schenkman et al. (18) demonstrated that HIIT with a heart rate of up to 85% of HRmax on the treadmill is feasible for PwPD and results insignificant greater improvements on the UPDRS (Unified Parkinson Disease Rating Scale) compared to low-frequency training (18). In contrast to Schenkman et al. (18), the present pilot study focuses specifically on cognitive outcomes. A study has now also been published on an even more intense form of HIIT, it compares HIIT at intensities of 90%–99% of maximum heart rate (HRmax) with continuous training (CT) at intensities of over 80% of HRmax, among other things, and examines sessions lasting 30 min or longer (19). This study uses very similar parameters.

Several studies have already examined endurance training using a bicycle ergometer in PwPD. A large-scale review and meta-analysis including 40 studies investigated the effects of various training modalities (20). These positive results have since been confirmed and expanded upon, with the exclusion of PwPD at Hoehn and Yahr stage IV (21), who had also been included in the present pilot study. However, the effects on cognitive performance were not analyzed in that study.

The current recommendations for physical therapy for people with PwPD do not comment on training on a bicycling ergometer (22). Nevertheless, there are studies that deal with HIIT on the bicycle ergometer and examine intervention times (e.g., 12 weeks or more) (18, 23).

The aim of this randomized pilot study was to investigate the effects of high-intensity interval training (HIIT) on various cognitive functions, such as reaction time and cognitive flexibility, in people with Parkinson's disease (PwPD), in order to lay the groundwork for a larger-scale RCT.HIIT is compared with CT of similar intensity on a cycle ergometer. We hypothesized that HIIT would lead to greater improvements in cognitive performance than CT because participants must repeatedly adapt to changing resistance during HIIT sessions.

## 2 Material and methods

### 2.1 Recruitment and participants

We prospectively recruited PwPD who were randomly assigned to two intervention groups—HIIT or CT. PwPD were recruited at the Lucerne Cantonal Hospital in Switzerland.

**Figure 1:**
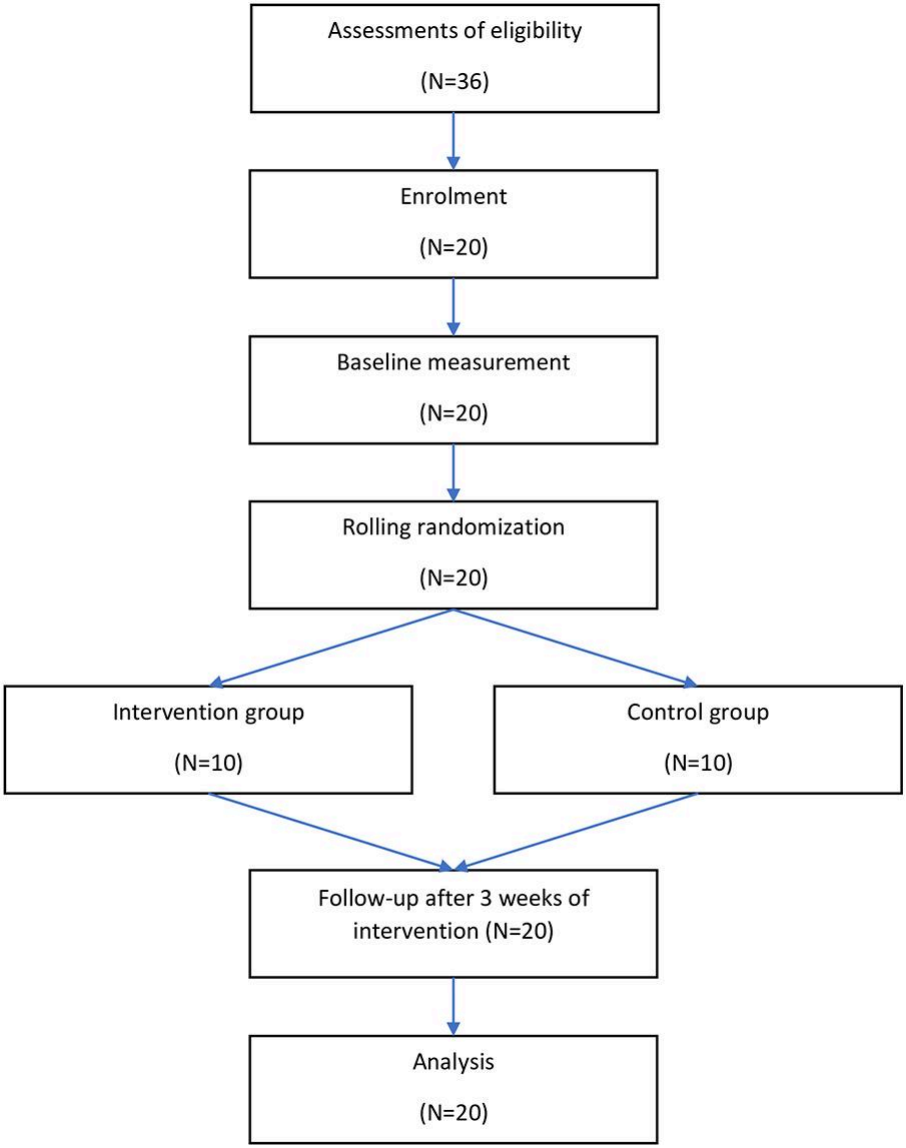
Flowchart.

PwPD were eligible for inclusion in the study if they had a diagnosis of PD as defined by the UK Parkinson's Disease Society Brain Bank Criteria (24) and were able to exercise on a bicycle ergometer for 30 min. Exclusion criteria were other brain organic disorders and concomitant cardiac disease. PwPD suffering from severe dementia were also excluded based on the Montreal Cognitive Assessment (MoCA). This is a screening procedure that examines a broad range of cognitive aspects (25). Individuals with a MoCa score below 12 points and those with Hoehn and Yahr Stage 5 were excluded. In addition, medication or settings of any deep brain stimulation were not allowed to be changed during the intervention phase. The study was approved by the local ethics committee (Ethics Committee for Northwestern and Central Switzerland EKNZ-2021-00339) and registered in the Swiss Registry of Human Research under the numbers HumRes55189 | SNCTP000004359 | BASEC2021-00339. Written informed consent was obtained from all participants, and the protocol adhered to the Declaration of Helsinki (Figure 1).

### 2.2 Procedure

At the start of the study, all Parkinson’s patients underwent cardiopulmonary exercise testing on a stationary bicycle to accurately determine their individual HRmax.

After consent PwPD were assigned consecutively to one of the two intervention groups. Randomization was concealed from the investigator. A randomization list was created by the Clinical Trial Unit—Central Switzerland using the Blockrand tool. Following baseline testing by the investigator, the subject was assigned to a group by a third person using the randomization list. All data on baseline, informed consent, assessments, each intervention, and study completion were documented in the online tool secuTrial®, which is maintained by an external company (Swiss Paraplegic Center, Lucerne). Assessments were done by the same investigator and done in best ON phase of PwPD (approximately 1 to ½ hours after medication intake).

### 2.3 Outcome measures

All outcomes were done at baseline, before start of training and then immediately after end of training.

### 2.4 Primary outcomes

#### 2.4.1 Trail making test (TMT)

The TMT provides information on visual search, scanning, processing speed, cognitive flexibility, and executive function (26). In addition, Part B not only captures higher cognitive functions, but is also more visually demanding because there are greater distances between the searched objects than in Part A. The TMT has already been used in other studies to assess cognitive function in people with PwPD (27–29). TMT A primarily measures processing speed, while TMT B also measures cognitive flexibility (30).

#### 2.4.2 Stroop tests

The Stroop Color-Word Interference Test measures the ability to prevent cognitive interference that occurs when processing one stimulus makes it difficult to process a second stimulus at the same time These occur when the processing of a particular stimulus makes the simultaneous processing of a second stimulus difficult (31). Three of the four sections were used for this study: Section 1, which measures processing speed; Section 3, for interference control; and Section 4, which measures cognitive flexibility. In all parts, the time the patient needs to name is measured. The Stroop test has also been used as a measurement parameter in studies involving people with Parkinson's disease.

### 2.5 Secondary outcomes

Spiroergometry, also known as cardiopulmonary exercise testing (CPET), is the gold standard for determining aerobic fitness in medicine. It provides a wealth of information, particularly about the function of the cardiovascular system and the respiratory tract, the neuromuscular system and metabolism, as well as the limits of physical performance. This makes it useful for the diagnosis, treatment, and prognosis of diseases (32). In this study, it is used as a baseline test for training control (Measuring device: Ganshorn PowerCub Ergo, Test report: Ramp test), in which intensity is calculated based on the highest heart rate (HRmax) achieved, as well as a repeat test after the intervention. Changes in maximum oxygen uptake capacity (VO2max) and maximum wattage are evaluated here.

#### 2.5.1 The Parkinson's disease questionnaire (PDQ-39)

The PDQ-39 is a self-report questionnaire consisting of 39 questions. It assesses Parkinson's-specific health-related quality of life in eight dimensions of quality of life (Mobility, Activities of daily living, Emotional well-being, Stigma, Social support, Cognition, Communication, Bodily discomfort). The rating scale ranges from 0 = never to 4 = always, The higher the score on the questionnaire, the more severe the impairment (33).

#### 2.5.2 Unified Parkinson disease rating scale (UPDRS), part II and III

This study uses the second and third parts of the Unified Parkinson's Disease Rating Scale (UPDRS). The second part of the UPDRS scale is a questionnaire that asks about motor aspects of everyday life. It consists of 13 items. The third part is an assessment of movements that are either read aloud or demonstrated by the examiner during the test. It contains a total of 18 items that are typically impaired in people with Parkinson's disease (34). On the UPDRS-III scale, the minimum clinically important difference (MCID) is five points (35).

#### 2.5.3 Freezing of gate questionnaire (FOG-Q)

The FOG-Q assesses the impairment of PwPD due to freezing of gait (FOG), regardless of falls. It measures the frequency of FOG, gait disturbances, and the association with clinical features related to walking and motor aspects. Six items are rated from 0 (no symptoms) to 4 (highest severity of symptoms), resulting in a maximum score of 0 and a minimum score of 24 (36).

#### 2.5.4 Mini BESTest

The Mini-BESTest is the short version of the Balance Evaluation Systems Test (BESTest). It consists of 14 tests in four areas of balance: anticipatory, reactive postural control, sensory orientation, and dynamic gate. The maximum score is 28 points (37).

### 2.6 Interventions

In this study, two different interventions were carried out. In both groups (intervention and control group) there were ten subjects who completed the program. For this pilot study, a sample size of 10 participants was selected for both the intervention and control groups. This sample size is based on recommendations for exploratory pilot studies, which are primarily intended to assess feasibility and estimate effect sizes and variability (38). With *n* = 10 per group, the standard deviation can be estimated with sufficient accuracy and allows for an initial assessment of the direction of the effect. One group performs HIIT training on a stationary bike three times a week for three weeks, while the other group trains for the same period of time using the continuous method. In total, the PwPD participants completed 9 training sessions across both groups. In both groups, the training sessions lasted a total of 30 min per unit. For safety reasons, the training was conducted on a stationary bike rather than on a treadmill, as is often the case in studies. This made it possible to include patients even in advanced stages of the disease. The benefits of this exercise device for PwPD have already been demonstrated in another study (39). All training sessions took place in the physical therapy department of Lucerne Cantonal Hospital. The training sessions were supervised by a physical therapist at all times.

#### 2.6.1 Intervention group: HIIT

In detail, the intervention was as follows: The warm-up included five minutes at 60% of HRmax. Subsequently, the interval training was performed and lasted twenty minutes. The intervals were performed for 30 s at 75% HRmax and 30 s at 90% HRmax in 20 cycles, respectively. This was followed by five minutes of cool-down, again at 60% HRmax. The pulse was permanently monitored by a pulse sensor in the chest strap of the Polar T31 (40).

#### 2.6.2 Control group: CT

The CT is a very well researched training method, which is used in different intensities. In general, an improvement in VO_2_max can be achieved with training (41). Low-intensity, moderate-intensity, and high-intensity training can be performed using duration methods (42). In this study we use high-intensity training.

The intervention with the CT consisted of a five-minute warm-up at 60% HRmax, a 20-minute workout at continuous 85% HRmax, and a subsequent five-minute cool-down at 60% HRmax. Again, the pulse was permanently monitored with the same heart rate monitor.

Both groups train at very similar overall intensities so that the results are primarily due to the different training modalities and less due to the intensity.

### 2.7 Statistical analysis

Statistical analysis was based on data drawn from the SecuTrail® tool and calculated using SPSS version 27. Descriptive statistics were used to represent baseline characteristics and outcome measure data. The main analyses were 2 × 2 Anova's to compare the outcomes between the two groups. Paired tests were then used to compare outcomes within groups. For all analyses, the significance level was set at *p* < 0.05. Participants who were unable to complete one of the tests were excluded from the calculations in this assessment, and the results table showed that fewer patients were included at this endpoint.

## 3 Results

There was no patient who dropped out of the study, i.e., all twenty subjects completed the initial assessment, the training and the final tests in full. The demographic and clinical characteristics are presented in Table 1. The primary and secondary outcomes are presented in Table 2.

**Table 1:** Demographic and clinical characteristics.

Characteristics	Intervention (*N* = 10)	Control group (*N* = 10)	Group difference (*p*-value)
Age	69,8 (± 9,72)	67,6 (± 7,6)	.427
Gender (m/f)	7/3	6/4	.639
Hoehn & Yahr	2,1 (± 0,87)	1,9 (± 0,57)	.753
MoCA	23,5 (± 7,14)	26,5 (± 2,41)	.314
Duration of disease in years	5,9 (± 5,88)	4,6 (± 3,47)	.621
Weight (kg)	81,1 (± 16,99)	74 (± 8,84)	.940
Size (cm)	171 (± 9,16)	173,2 (± 9,42)	.595

The distribution of subjects across groups is presented in the table. Mean values and standard deviations are shown in parentheses. Group differences were analyzed using Mann–Whitney U tests for non-parametric data and *χ*² tests for categorical data.

**Table 2:** Primary and secondary outcomes.

Assessment	Group		At the beginning of the study			At the end of the study			T-Test	2 × 2 Anova		
		N	M	SD±	SEM	M	SD±	SEM	sig. 2-sided	F	P	_p_ *η* ^2^
MiniBest	Continuous method	10	21.70	4.029	1.274	24.10	3.573	1.130	0.000	0.110	0.744	0.006
	HIIT	10	19.10	5.820	1.841	21.70	6.308	1.995	0.000			
MiniBest Subscore 1	Continuous method	10	4.60	1.506	0.476	5.00	0.943	0.298	0.168	1.301	0.269	0.067
	HIIT	10	4.20	1.476	0.467	5.10	1.287	0.407	0.029			
MiniBest Subscore 2	Continuous method	10	4.50	1.509	0.477	5.10	1.287	0.407	0.111	0.214	0.649	0.012
	HIIT	10	3.50	2.121	0.671	3.90	2.079	0.657	0.168			
MiniBest Subscore 3	Continuous method	10	5.30	0.949	0.300	5.30	0.675	0.213	1.000	0.878	0.361	0.047
	HIIT	10	4.80	1.398	0.442	5.20	1.317	0.416	0.223			
MiniBest Subscore 4	Continuous method	10	7.30	2.163	0.684	8.70	1.567	0.496	0.034	0.637	0.435	0.034
	HIIT	10	6.60	2.319	0.733	7.50	2.718	0.860	0.010			
PDQ39	Continuous method	10	28.80	18.772	5.936	23.90	19.058	6.027	0.113	0.421	0.525	0.023
	HIIT	10	26.80	24.903	7.875	24.20	23.266	7.357	0.265			
FogQ	Continuous method	10	5.50	5.759	1.821	5.30	5.982	1.892	0.678	0.067	0.799	0.004
	HIIT	10	7.00	6.018	1.903	6.60	4.971	1.572	0.534			
UPDRS II	Continuous method	10	10.50	4.927	1.558	8.90	4.630	1.464	0.006	0.276	0.605	0.015
	HIIT	10	13.40	9.216	2.914	11.50	8.898	2.814	0.000			
UPDRS III	Continuous method	10	20.00	8.000	2.530	16.20	7.843	2.480	0.004	0.410	0.530	0.022
	HIIT	10	29.80	19.066	6.029	25.10	17.045	5.390	0.001			
TMT A	Continuous method	10	00:44	00:25	00:07	00:37	00:13	00:04	0.141	0.282	0.603	0.016
	HIIT	9	01:20	01:41	00:33	01:08	01:13	00:24	0.269			
TMT B	Continuous method	9	01:51	00:54	00:18	02:08	01:20	00:26	0.285	**12** **.** **716**	**0**.**003**	**0**.**459**
	HIIT	8	03:14	02:11	00:46	02:05	01:49	00:38	0.010			
Stroop I	Continuous method	10	00:35	00:06	00:01	00:35	00:05	00:01	0.867	1.165	0.295	0.061
	HIIT	10	00:58	01:03	00:19	00:49	00:38	00:12	0.294			
Stroop III	Continuous method	10	01:43	01:13	00:23	01:17	00:25	00:08	0.261	0.462	0.506	0.028
	HIIT	8	01:32	00:41	00:14	01:23	00:37	00:13	0.019			
Stroop IV	Continuous method	10	01:22	00:12	00:04	01:19	00:12	00:04	0.608	0.578	0.459	0.037
	HIIT	7	01:21	00:22	00:08	01:11	00:07	00:02	0.338			
VO_2_max	Continuous method	10	1.52	0.562	0.178	1.77	0.603	0.191	0.010	4.148	0.057	0.187
	HIIT	10	1.79	0.556	0.176	1.80	0.600	0.190	0.865			
MaxWatt	Continuous method	10	131.10	44.273	14.000	143.90	49.970	15.802	0.003	0.194	0.665	0.011
	HIIT	10	126.10	43.247	13.676	141.50	55.354	17.504	0.012			
HRmax	Continuous method	10	129	15.46	4.890	127.9	14.26	4.510	0.705	1.372	0.257	0.071
	HIIT	10	127.8	18.75	5.929	130.7	18.08	5.718	0.878			

N, number of subjects; M, mean; SD, standard deviation; SEM, standard error of the mean; F, *F*-test; P, *p*-value; pη², partial eta-squared.

The significant differences between groups are highlighted in bold.

### 3.1 Primary outcomes

For TMT A there was no significant change in the CT group (*n* = 10, *p* = 0.141). In the HIIT group (*n* = 9), the effect was also not significant at *p* = 0.269. There was also no significant interaction effect between groups at *p* = 0.603, F is 0.282 and _p_*η*^2^ = 0.016. One participant in the HIIT group was unable to perform this test because he did not understand the task. After the intervention, this participant was able to complete the test with an increased time requirement. This value was not considered in the evaluation.

In TMT B, this test could not be performed in two subjects of the HIIT group and one subject of the control group, due to their cognitive deficits. In the CT group (*n* = 9) there was no significant change *p* = 0.285. In the HIIT group (*n* = 8) the effect was significant at *p* = 0.010. Training in the HIIT group leads to a significant improvement in test results in TMT-B compared to CT. There is a significant interaction effect between groups with *p* = 0.0.003, F = 12.716 and _p_*η*^2^ = 0.459.

For the Stroop I in the CT group (*n* = 10) there was no significant change *p* = 0.867. In the HIIT group (*n* = 10) the effect was also not significant with *p* = 0.294. There is no significant interaction effect between the groups with *p* = 0.295, F = 1.165 and _p_*η*^2^ = 0.061.

For Stroop III. Two individuals from the HIIT group were unable to perform this test at the first examination. However, one of the two participants was able to perform the test after HIIT training for three weeks. This value was not considered in the analysis. In the CT group (*n* = 10) there was no significant change *p* = 0.261. In the HIIT group (*n* = 8) the effect was significant at *p* = 0.019. There is no significant interaction effect between groups with *p* = 0.506, F = 0.462 and _p_*η*^2^ = 0.028.

For Stroop IV three subjects from the HIIT group were unable to perform this test before and after the intervention. In the other group, all subjects were able to perform the test. In the CT group (*n* = 10) there was no significant change *p* = 0.413. In the HIIT group *n* = 7) the effect was also not significant with *p* = 0.338. There is no significant interaction effect between groups with *p* = 0.459, F = 0.578 and _p_*η*^2^ = 0.037.

### 3.2 Secondary outcomes

For all secondary outcomes, all subjects completed the tests at baseline and at completion. The Mini BESTest revealed no significant differences between the two groups (*p* = 0.744, F = 0,110 and _p_*η*^2^ = 0.006). The same was true for the other tests: PDQ 39 showed no differences with *p* = 0.525, F = 0.421 and _p_*η*^2^ = 0.023, Fog-Q (*p* = 0.799, F = 0.067 and _p_*η*^2^ = 0.004), UPDRS II (*p* = 0.605, F = 0.276 and _p_*η*^2^ = 0.015), and UPDRS III (*p* = 0.530, F = 0,410 and _p_*η*^2^ = 0.022) all ended without differences between the groups.

However, both the intervention and control groups showed highly significant improvements between the two measurement points in the Mini BESTest (control group *p* < 0.001 and intervention group *p* < 0.001), in the UPDRS II scale (control group *p* = 0.006, intervention group *p* < 0.001) and in the UPDRS III scale (control group *p* = 0.004, intervention group *p* = 0.001). There was no difference in VO2max between the groups (*p* = 0.057, F = 4.418 and _p_*η*^2^ = 0.187), with the control group achieving a significant improvement of *p* = 0.010, while the intervention had no effect (*p* = 0.865). There was no difference in the maximum wattage achieved by the groups in spiroergometry (*p* = 0.665, F = 0,194 and _p_*η*^2^ = 0.011), but both groups showed a significant improvement between baseline and end (control group *p* = 0.003, intervention group *p* = 0.012).

## 4 Discussion

The purpose of this pilot randomized study was to investigate whether HIIT on a cycle ergometer leads to greater cognitive improvements individuals with PD compared with CT. The main findings partially support our hypothesis. While no significant group-by-time interactions were observed for most cognitive outcomes, HIIT resulted in significant within-group improvements in executive function such as cognitive flexibility and inhibitory control, as reflected by better performance in TMT-B and Stroop III. In contrast, CT did not lead to significant changes in these cognitive domains.

TMT-B and Stroop III both assess higher-order executive functions, including cognitive flexibility, set shifting, and inhibitory control (43, 44). These cognitive domains are particularly vulnerable in PwPD (45), and are strongly associated with functional independence (46). The observed improvements in the HIIT group suggest that short, alternating bouts of high and moderate intensity may provide a stronger cognitive stimulus than continuous exercise at comparable overall intensity. A possible mechanism is the repeated need to rapidly adapt to changing workloads during HIIT, which may engage frontostriatal circuits and promote neuroplastic adaptations more effectively than steady-state exercise (47, 48). This interpretation aligns with previous work indicating that cognitively demanding physical tasks may amplify exercise-induced benefits on executive functioning (49).

The absence of significant interaction effects between groups likely reflects the limited statistical power of this pilot study and the small sample size, compounded by missing data due to participants being unable to complete certain cognitive tests. However, this was also not the aim of this pilot study, being purely exploratory in nature. Nevertheless, the consistent pattern of within-group improvements favoring HIIT on TMT-B and Stroop III supports the feasibility and potential superiority of HIIT for targeting executive functions in PwPD. Our findings extend prior work by (18), who demonstrated the feasibility and motor benefits of HIIT in PwPD, by highlighting possible cognitive advantages of this training modality. Interestingly, no significant effects were observed for TMT-A or Stroop I and IV. TMT-A primarily reflects processing speed and visual scanning rather than executive control, which may explain its lower sensitivity to training-related changes over a short intervention period. Similarly, Stroop I represents basic colour naming, whereas Stroop IV requires more complex task switching. The lack of improvement in Stroop IV may be attributable to floor effects, as several participants in the HIIT group were unable to complete this task, indicating that it may have exceeded their cognitive capacity. Across all cognitive assessments, we observed improvements achieved with both interventions. 29 had already demonstrated improvements in the aforementioned outcomes with moderate training, which is consistent with our findings here.

Regarding secondary outcomes, both groups demonstrated significant improvements in balance (Mini-BESTest) and motor function (UPDRS II and III), with no between-group differences. These results are consistent with previous literature showing that aerobic exercise, regardless of the exercise intensity and training type, can improve motor symptoms and functional mobility in PwPD (50, 51). However, it appears that the MCID of 5 points on the UPDRS scale was narrowly missed, with average improvements of 3.8 points (CT) and 4.7 points (HIIT).

The comparable training progress in both groups suggests that high-intensity endurance training on a stationary bike is just as effective as HIIT in terms of motor performance characteristics over a short training period. Contrary to expectations, VO₂max improved significantly only in the endurance training group. This finding may be explained by the short intervention period of just 3 weeks. Other studies with longer intervention periods have shown that HIIT can have a greater effect on VO₂max (19). Although HIIT has been associated with superior mitochondrial adaptations in other populations (52), the short intervention duration (three weeks) and relatively conservative HIIT intensity (75%–90% HRmax) may have limited measurable aerobic gains in this group. No significant changes were observed in quality of life or freezing of gait. These outcomes may require longer intervention periods to show meaningful change and are also influenced by multiple psychosocial and disease-related factors beyond physical training alone. Improvements in these areas achieved through intensive training over a longer intervention period have been demonstrated in other studies (53).

Several limitations should be acknowledged. First, this was a small pilot study with limited statistical power, increasing the risk of type II error and reducing generalizability. Second, cognitive assessments were constrained by participants’ abilities, resulting in missing data and unequal group sizes for some outcomes. This is exacerbated by the fact that there were already differences in cognitive abilities at baseline, as evidenced by a wider range of scores on the MoCA in the HIIT group compared to the CT group. Third, the intervention period was relatively short, which may have been insufficient to elicit larger cognitive or aerobic adaptations. Finally, although assessors were kept consistent across evaluations to reduce inter-rater variability and improve scoring reliability, blinding was not implemented, introducing potential assessment bias.

To conclude, this pilot randomized study demonstrates that HIIT on a cycle ergometer is feasible and well tolerated in PwPD and may preferentially enhance executive function compared with CT. Given the importance of cognitive flexibility and inhibitory control for daily functioning and fall prevention, HIIT could represent a valuable addition to rehabilitation programs; however, studies with follow-up measurements are needed to assess the sustainability of the intervention. Future studies with larger sample sizes, longer intervention durations, and follow-up assessments are warranted to confirm these preliminary findings and to explore underlying neurobiological mechanisms. Incorporating neuroimaging or biomarkers of neuroplasticity may further clarify how different aerobic training modalities influence cognitive outcomes in Parkinson's disease.

## Data Availability

The original contributions presented in the study are included in the article/Supplementary Material, further inquiries can be directed to the corresponding author.
